# Age-Related Changes in Plasma Extracellular Vesicle Characteristics and Internalization by Leukocytes

**DOI:** 10.1038/s41598-017-01386-z

**Published:** 2017-05-02

**Authors:** Erez Eitan, Jamal Green, Monica Bodogai, Nicolle A. Mode, Rikke Bæk, Malene M. Jørgensen, David W. Freeman, Kenneth W. Witwer, Alan B. Zonderman, Arya Biragyn, Mark P. Mattson, Nicole Noren Hooten, Michele K. Evans

**Affiliations:** 1 Laboratory of Neurosciences, 251 Bayview Boulevard, Baltimore, MD 21224 USA; 2 Laboratory of Epidemiology and Population Science, 251 Bayview Boulevard, Baltimore, MD 21224 USA; 3 Laboratory of Molecular Biology and Immunology, National Institute on Aging, National Institutes of Health, 251 Bayview Boulevard, Baltimore, MD 21224 USA; 4 Department of Clinical Immunology, part of EVSearch.dk, Aalborg University Hospital, Aalborg, Denmark; 5 Department of Molecular and Comparative Pathobiology, and Department of Neurology, The Johns Hopkins University School of Medicine, Baltimore, MD USA

## Abstract

Cells release lipid-bound extracellular vesicles (EVs; exosomes, microvesicles and apoptotic bodies) containing proteins, lipids and RNAs into the circulation. Vesicles mediate intercellular communication between both neighboring and distant cells. There is substantial interest in using EVs as biomarkers for age-related diseases including cancer, and neurodegenerative, metabolic and cardiovascular diseases. The majority of research focuses on identifying differences in EVs when comparing disease states and matched controls. Here, we analyzed circulating plasma EVs in a cross-sectional and longitudinal study in order to address age-related changes in community-dwelling individuals. We found that EV concentration decreases with advancing age. Furthermore, EVs from older individuals were more readily internalized by B cells and increased MHC-II expression on monocytes compared with EVs from younger individuals, indicating that the decreased concentration of EVs with age may be due in part to increased internalization. EVs activated both monocytes and B cells, and activation of B cells by LPS enhanced EV internalization. We also report a relative stability of EV concentration and protein amount in individual subjects over time. Our data provide important information towards establishing a profile of EVs with human age, which will further aid in the development of EV-based diagnostics for aging and age-related diseases.

## Introduction

Extracellular vesicles (EVs) are nano-sized (30–400 nm) membranous vesicles that are secreted by a variety of cell types into the circulation and other bodily fluids. In the blood, there are at least three types of EVs: exosomes released by the fusion of multivesicular bodies with the plasma membrane, microvesicles released by budding of small segments of the plasma membrane, and apoptotic bodies released from dying cells^1, 2^. The size, density, and cargo of these different types of EVs overlap, making it difficult to determine the type of EVs isolated from cell culture and body fluid samples^3^. EVs contain proteins, RNA and bioactive lipids and have biological and physiological roles in both homeostatic and pathological conditions^1, 2^. For example, EVs are important for immune responses, stem cell differentiation and vascularization, and have been found to play important roles in tumor development and metastasis^2, 4^. Furthermore, recent findings suggest that EVs play critical roles in a variety of age-related chronic diseases including neurodegenerative diseases (e.g., Alzheimer’s disease), metabolic diseases, and cardiovascular disease^5^.

Circulating EVs are thought to be released by platelets, erythrocytes and endothelial cells^6, 7^, but EVs from neurons, adipocytes and several other cell types can also be detected^8–10^. Given that a variety of cell types secrete EVs into the circulation, EVs have recently been identified as attractive biomarkers of tissue-specific diseases. Cancer cells *in vitro* have been shown to secrete more EVs than non-transformed cells^11^. Increased concentration of EVs has also been reported in the blood of autoimmune patients^10, 12^, but this is not a general disease effect as no changes in EV concentration were detected in plasma samples of Alzheimer’s disease patients^8^.

Emerging roles for EVs in age-related diseases suggest that EVs have diagnostic and therapeutic potential. To enable efficient diagnostic usage, EVs must be more broadly characterized in human populations. However, few studies have focused on characterizing EVs in community-dwelling individuals, who are non-hospitalized and not acutely ill. There are no data concerning the amount, sizes and molecular composition of EVs during normal aging. One paper examined miRNA profiles from plasma and peripheral blood mononuclear cells (PBMCs) from male and female donors^13^. Interestingly, miRNA profiles differed considerably between EVs and PBMCs, further verifying their diverse cellular origin and the importance of examining EVs specifically. The morphology of plasma EVs was also recently described for 5 healthy male donors by cryo-transmission electron microscopy^6^.

The accumulating evidence that EVs play a role in age-related disease, and the potential use of EVs as diagnostic and prognostic markers, led us to examine whether EVs change with age in humans. Here, we analyzed EVs in a cross-sectional and longitudinal study and found an age-related decrease in EVs concentration and altered uptake by immune cells. Establishing a profile of EVs with human age will further aid in the development of diagnostics and therapies using EV-designed technologies for age-related diseases.

## Results

### EV Concentration Changes with Age

**Figure 1:**
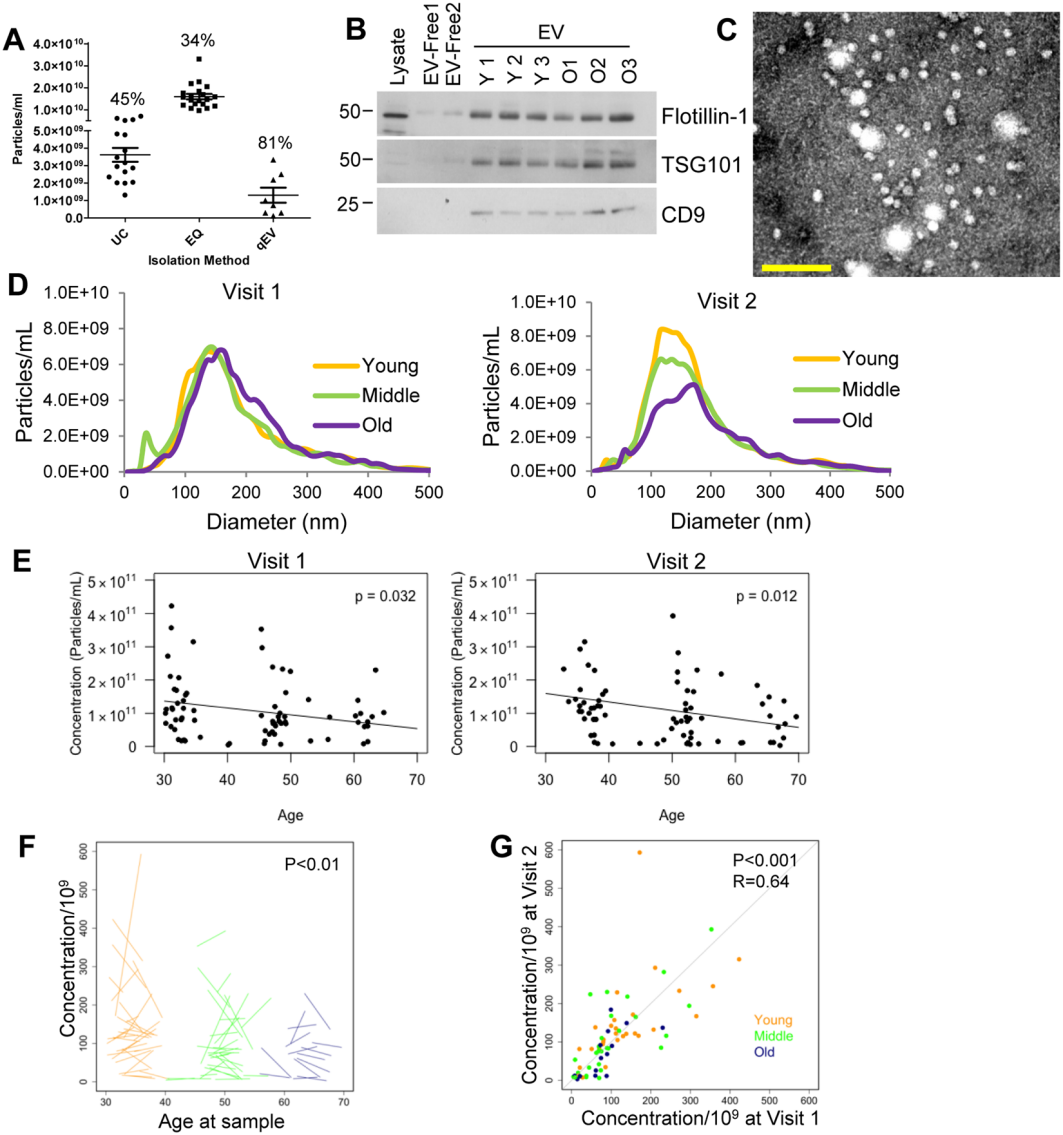
Plasma EV Concentration decreases with human age. (**A**) Comparision of different EV isolation techniques. The same plasma sample was divided into 46 equal aliquots of 200 μl and EVs were isolated by different methods. Samples were isolated by ultracentrifugation (UC, n = 17), ExoQuick (EQ, n = 20) and size exclusion chromotography (qEV, n = 9). Particle concentration was quantifed by NTA and the coefficient of variance was calculated and indicated for each group. (**B**) Plasma EV samples (5 μg) from three young (Y1- Y3), three old (O1–O3) individuals, two EV-depleted plasma fractions (EV-free) from subjects Y1 and O1, and neuronal cell lysate were lysed and analyzed by SDS-PAGE followed by immunoblotting with antibodies against the indicated EV markers. The experiment was repeated four times. (**C**) EVs of typical morphology and size range of 50 to 200 nm were observed by electron microscopy. Scale bar = 200 nm (**D**) NTA was used to determine the size distribution of plasma EVs. Each sample was averaged for the young (n = 30), middle aged (n = 30), and old (n = 14) groups for both time points. (**E**) NTA was used to determine the EV plasma concentration for each individual of the 74 participants in cross-sectional analyses of the two different time points. P value was determined by linear mixed-model regression. (**F**) The changes in concentration overtime are represented by a spaghetti plot where the start of each line represents the starting EV plasma concentration and the end of the line represents the concentration at the last time point. (**G**) The concentration at time point 1 and time point 2, are plotted on the x and y axis. The similarity between an individual’s EV concentrations at the two time points were analyzed by Pearson correlation.

In order to examine the relationship between age and EVs circulating in the blood, we isolated plasma EVs from a sub-cohort of the Healthy Aging in Neighborhoods of Diversity across the Life Span (HANDLS) study, which is a longitudinal, epidemiologic study of aging. This sub-cohort consisted of 30 young individuals (30–35 years), 30 middle-aged individuals (40–55 years), and 14 old individuals (55–64 years), who had contributed plasma at two different time points (Visit 1 and Visit 2) approximately 5 years apart (mean = 4.60 ± 1.04 years) to allow both cross-sectional and longitudinal analyses. The participants in each age group were matched by race and sex (Table 1). This repeated measures approach enhances the statistical power of the analysis. The EVs were isolated from plasma using a precipitation approach^14–16^. We chose this method after careful consideration and evaluation, since differential ultracentrifugation procedures were not feasible for a large number of samples, and because this method provided more reproducible data than either differential ultracentrifugation or size exclusion columns (Fig. 1A). This method would also allow us to further characterize EVs using a variety of experimental approaches.

**Table 1:** Demographic and clinical characteristics of the EV cohort.

Characteristics	Visit	Young	Middle Aged	Old	P-value
N		30	30	14	
Male n (% Total)		16 (53%)	17 (57%)	6 (43%)	0.691
African Americans n (% Total)		14 (47%)	15 (50%)	9 (64%)	0.543
Whites n (% Total)		16 (53%)	15 (50%)	5 (36%)	
Age	1	32.30 ± 1.43	47.40 ± 2.69	61.09 ± 2.45	<0.001
	2	36.97 ± 1.75	52.00 ± 2.50	65.62 ± 2.51	<0.001
BMI	1	26.08 ± 4.94	25.24 ± 5.10	28.74 ± 4.76	0.098
	2	27.62 ± 6.27	25.86 ± 5.24	27.78 ± 4.79	0.399
Weight, kg	1	78.57 ± 15.38	73.17 ± 16.58	78.79 ± 13.42	0.337
	2	82.77 ± 16.67	74.90 ± 17.34	75.71 ± 14.46	0.158
Smoker n (% Total)	1	10 (38%)	16 (59%)	2 (14%)	0.020
	2	10 (34%)	11 (38%)	2 (14%)	0.276

Age, BMI, and weight represent the mean ± SD. Pearson’s chi-squared tests were used to analyze differences among the age groups for gender, race, and current smoking status. Smoking status was missing for 7 individuals at visit 1 and 2 individuals at visit 2. One-way ANOVAs were used to analyze differences among the age groups for BMI, weight and age.

The presence of EVs in our plasma samples was examined according to the guidelines of the International Society of Extracellular Vesicles^3^. First, isolated EVs from young and old individuals were analyzed by immunoblotting with antibodies against documented EV markers including CD9, Flotillin-1, and TSG101 (Fig. 1B). All EV markers were enriched in the EV samples compared to the EV-depleted plasma (Fig. 1B). EVs were also confirmed by performing electron microscopy and nanoparticle tracking analysis (NTA) on isolated EVs (Fig. 1C,D). The electron microscopy image shows clear rounded membrane vesicles in a size range of 50–250 nm, which is characteristic of EVs (Fig. 1C). The NTA data also confirmed a similar size distribution with a peak at 150 nm (Fig. 1D). EV size variations between electron microscopy and NTA have also been reported previously^17^.

We validated the accuracy of the NTA measurements by running a mix of 50 nm and 100 nm silica beads before running each set of EV samples. The error in the determination of the bead concentration was always below 10%. Moreover, we measured several EV samples for their size and concentration 4 independent times, and the coefficient of variance for concentration and size were below 20%. These measurements show that NTA is accurate and reproducible, thus we were able to examine the size distribution and concentration of the plasma EV samples from our cohort of individuals that were young, middle-aged and old in the two different visits. A typical size distribution of EVs was observed in both visits (Fig. 1D), but there were no significant alternations in the average size of EVs between the age groups. The EV sizes were also divided into 10 and 50 nm diameter groups to analyze differences and also grouped into larger size categories based on size as either exosomes (~50 nm–150 nm) or microvesicles (~150–350 nm) (Fig. S1). However, no consistent and significant changes in EV size were observed with age using any of these analysis strategies.

Although little is known about the concentration of EVs in the blood, recent reports show that EV blood concentration can change in ovarian cancer and lung cancer patients^18, 19^. Therefore, in addition to size we also wanted to determine whether the concentration of EVs changes with age. EVs from the 74 individuals at the two visits were analyzed by NTA and linear mixed model regression. We found a significant (visit 1*P* = 0.032 and visit 2*P* = 0.012) decrease in EV concentration with age after adjusting for BMI (Fig. 1E). There were no significant interactions between age and BMI.

We used linear mixed model regression to examine whether EV concentration is related to demographic and anthropometric measures. BMI (*P* = 0.229 for visit 1 and *P* = 0.035 for visit 2) and smoking status (*P* = 0.287 for visit 1 and *P* = 0.002 for visit 2) were significantly associated with EV concentration only at visit 2 (Fig. S2 and Table 2). There were no significant changes in EV concentration with sex or race (Table 2).

**Table 2:** EV concentrations by demographics.

	Visit			P Value
		Male	Female	
Sex	1	1.10E + 11 ± 0.93E + 11	1.08E + 11 ± 0.89E + 11	0.928
	2	1.09E + 11 ± 0.87E + 11	1.09E + 11 ± 1.16E + 11	0.796
		Whites	African American	
Race	1	1.01E + 11 ± 0.92E + 11	1.17E + 11 ± 0.90E + 11	0.433
	2	1.05E + 11 ± 1.28E + 11	1.19E + 11 ± 0.69E + 11	0.542
		Smoker	Non-Smoker	
Smoking	1	1.20E + 11 ± 1.02E + 11	0.96E + 11 ± 0.79E + 11	0.287
	2	1.66E + 11 ± 1.30E + 11	0.89E + 11 ± 0.75E + 11	0.002

Average EV concentrations ± SD are indicated for different demographics. *P* values for likelihood ratio tests from linear mixed model regression accounting for matching by race and sex.

### Longitudinal Changes in EVs

We chose individuals for our cohort that contributed two plasma samples approximately 5 years apart (mean = 4.60 ± 1.04 years) to examine longitudinal changes. The plasma concentration of EVs also significantly decreased longitudinally from visit 1 to visit 2 (Fig. 1F). To address whether an individual’s EV concentrations were maintained between the two visits, we examined the correlation. Interestingly, we observed a significant correlation (r = 0.64; *P* < 0.001) of EV concentration between visit 1 and visit 2, indicating a strong relationship between an individual’s EV concentration over time (Fig. 1G). These data suggest that although there are longitudinal changes in EV concentration with time, there is a high degree of similarity in the two different time points for each individual.

### Increased Internalization of Aged Extracellular Vesicles by B Cells

**Figure 2:**
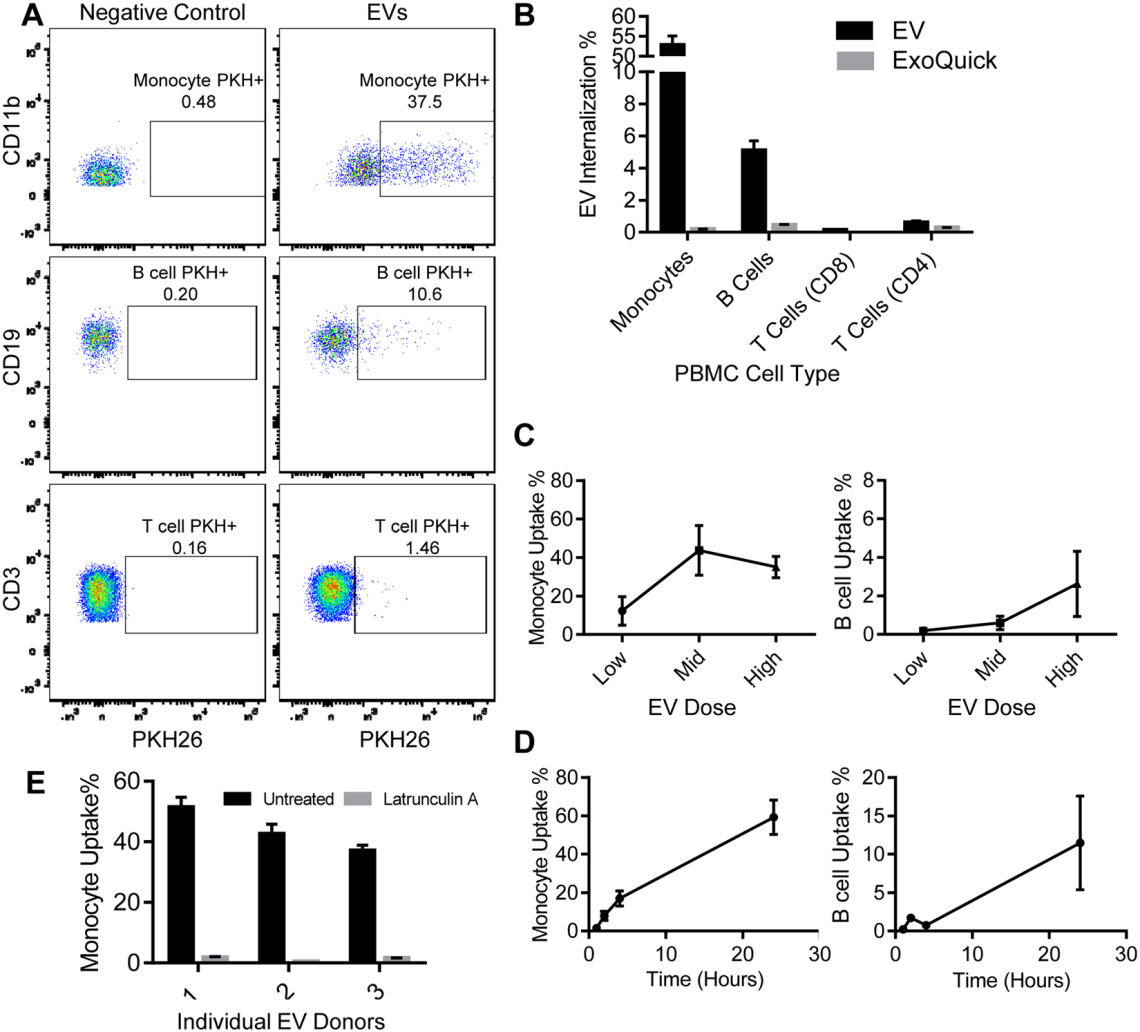
PBMC EV internalization Assay. (**A,B**) Plasma EVs (1.20^E+09^) were incubated with PBMCs for 24 hours and then analyzed for cell surface markers for monocytes (CD11b^+^), B cells (CD19^+^), and T cells (CD3^+^) and for EV internalization (PKH^+^) by FACS. Representative FACS dot plots showing the gating strategy is shown. The percentage of cells that internalized the PKH labelled EVs is indicated. (B) As a negative control, a 1:4 ExoQuick and PBS mixture was also labelled with PKH. In this representative experiment CD4^+^ and CD8^+^ T cells were examined separately. (**C**) A low (3.00^E+08^ particles), medium (6.00^E+08^ particles) and high dose (1.20^E+09^ particles) of PKH-labeled EVs were added to PBMCs and then analyzed by FACS. The histogram represents the mean of 3 EV donors + SEM. (**D**) PBMCs were incubated with EVs (3.00^E+08^ particles) for 1, 2, 4, and 24 hours and then analyzed by FACS. Note that the y-axes are different between monocytes and B cells in C-D. The histogram represents mean from 2 EV donors added separately to 2 PBMC donors + SEM. (**E**) PBMCs were incubated with Latrunculin A (1 ng/mL) for 30 min, and then a low dose of EVs were added to the untreated and treated PBMCs. The histogram represents the mean of two technical repeats for each EV donor + SEM.

The decrease in EV concentration with age we observed could be due to either a lower level of release of vesicles into the circulation or an increase in internalization of vesicles into cells. Vesicles in the circulation interact with a variety of circulating cells including monocytes, B cells, and T cells^20^. To examine whether decreased concentration is due to an increase in internalization by circulating cells, we established a FACS-based assay to measure the internalization of EVs by PBMCs. Since little is known about what circulating cells internalize EVs, we first determined what cell type internalizes EVs. EVs from different individuals were labeled with a fluorescent membrane dye PKH26, excess dye was removed by a size column with an average 72.86% ± 7.61% recovery of the EVs. Due to slight variation in EV recovery, the EV concentration was then recalculated using NTA. PBS and precipitation agent with PKH26 (and without EVs) was transferred through the size column and used as a negative control. PKH26-labeled EVs were incubated with freshly isolated human PBMCs for 24 hours and then analyzed using specific markers for monocytes (CD11b^+^CD14^+^ or CD11b^+^CD14^+^CD15^−^), B cells (CD19^+^), and T cells (CD3^+^ or CD4^+^/CD8^+^) using FACS. EVs were internalized by monocytes and B cells, but the internalization of EVs by T cells was comparable to the negative control despite being the most abundant PBMC in the circulation (Fig. 2A,B).

The percentage of monocytes and B cells with detectable internalized EVs was dose- and time- responsive in the assay (Fig. 2C,D). While the internalization by B cells increased from the low to middle to high doses of EVs, monocytes showed saturation in internalization around the middle EV dose (Fig. 2C). There was an increase in EV internalization in both cell types up to 24 hours (Fig. 2D).

To determine if this assay was measuring passive or active internalization by cells, we inhibited endocytosis in monocytes with latrunculin A. Latrunculin A almost completely abolished EV uptake in monocytes (Fig. 2E). Given that latrunculin A inhibits endocytosis, these data suggest that one mechanism of internalization for EVs by monocytes is through endocytosis.

**Figure 3:**
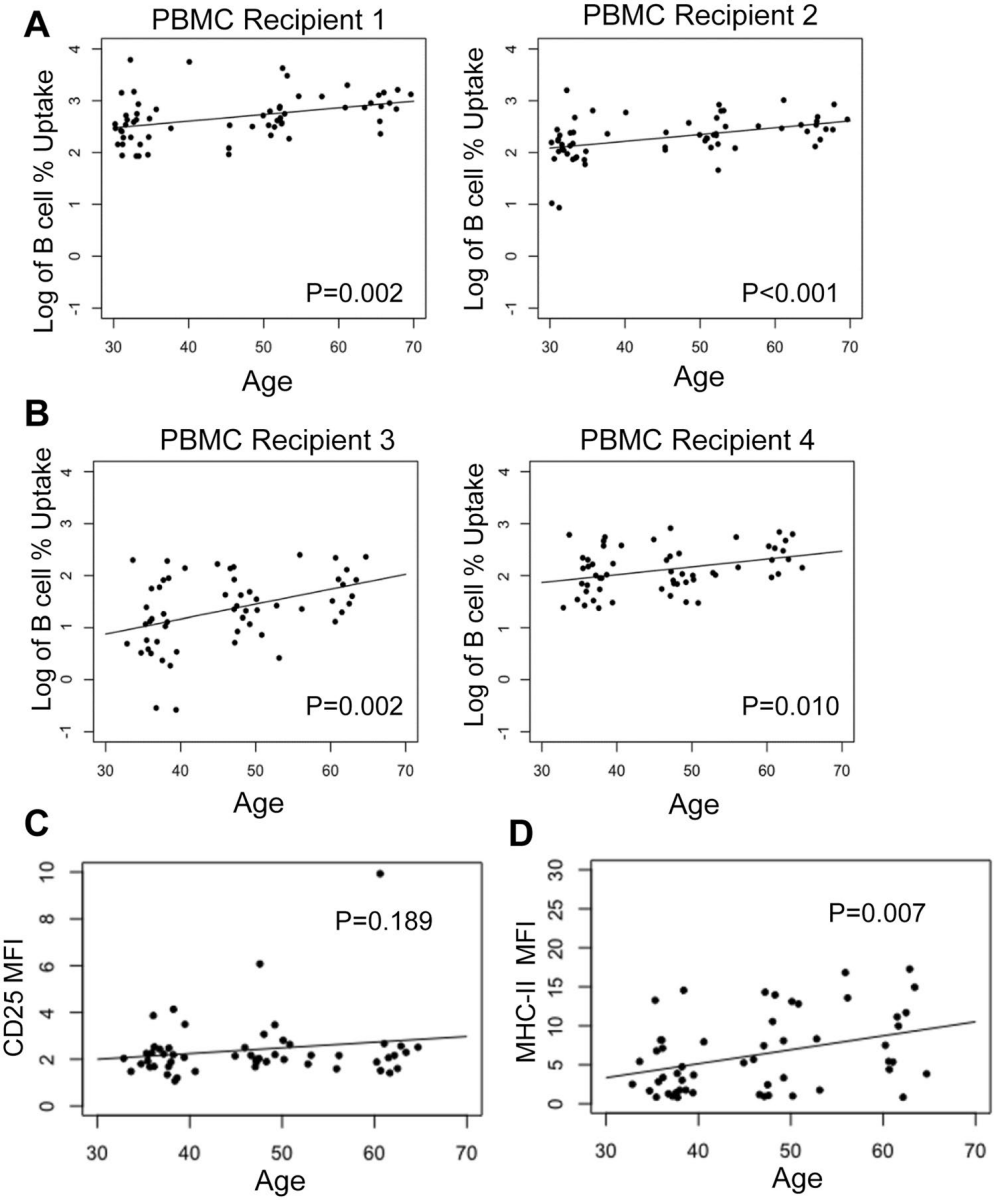
B Cells internalize older EVs more readily. (**A**) EV plasma samples (n = 62; 2.00^E+08^ particles) from young, middle-aged and older individuals were incubated with 1 old (1) and 1 young (2) PBMC recipient. (**B**) In a separate experiment, EV plasma samples (n = 57) from young, middle-aged and older individuals were incubated with a different old (3) and young (4) PBMC recipient. In both experiments, EVs were incubated with PBMCs for 24 hours and then anayzed by FACS. The points are the actual data (log transformed) and the line is from the model accounting for matching. (**C**) CD25 Mean Fluorescence Intensity (MFI) in B cells that interacted with EVs (CD19^+^PKH^+^CD25^+^) was plotted against the EV donor age. (**D**) MHC-II MFI in monocytes that interacted with EVs (CD11b^+^PKH^+^MHC-II^+^) was plotted against EV donor age. The graphs in C-D are 57 EV donors that were incubated with PBMC recipient 3. For all graphs, linear mixed model regression was used to analyze the relationships accounting for both sex and race matching and *P* values are indicated.

To test whether the decreased EV concentration with age may be due to increased internalization by circulating cells, we evaluated the internalization of isolated EVs from the ~74 young, middle-aged and old individuals (see above) by our FACS-based assay. We chose a lower dose of EVs (see Fig. 2C) as this would enable us to observe maximal differences in internalization by different EV donors. In four different PBMC recipients, B cells internalized EVs from older individuals more readily than EVs from younger individuals (Fig. 3A,B). In general, monocyte internalization of EVs did not change dramatically with EV donor age (Fig. S3). These data suggest that, in part, the decreased circulating EV concentration with age we observed may be due to an increase in internalization of EVs with age in B cells.

The effect of EV internalization on B cell and monocyte activation was examined by measuring the expression of the general activation markers CD25 for B cells and MHC-II for monocytes and also the costimulatory/activation marker CD80 for some of our studies. The age of the EV donor did not affect B cell activation (Fig. 3C and Fig. S4), but EVs from older donors increased the expression of MHC-II on monocytes more than EVs from young individuals (Fig. 3D and Fig. S4). Therefore, EV donor age affects EV internalization by B cells, but not B cell activation as reflected by CD25 expression. However, EV donor age affects monocyte activation, but not internalization by monocytes, indicating that EVs from older individuals might induce functional changes in monocytes despite a lack of difference in uptake.

**Figure 4:**
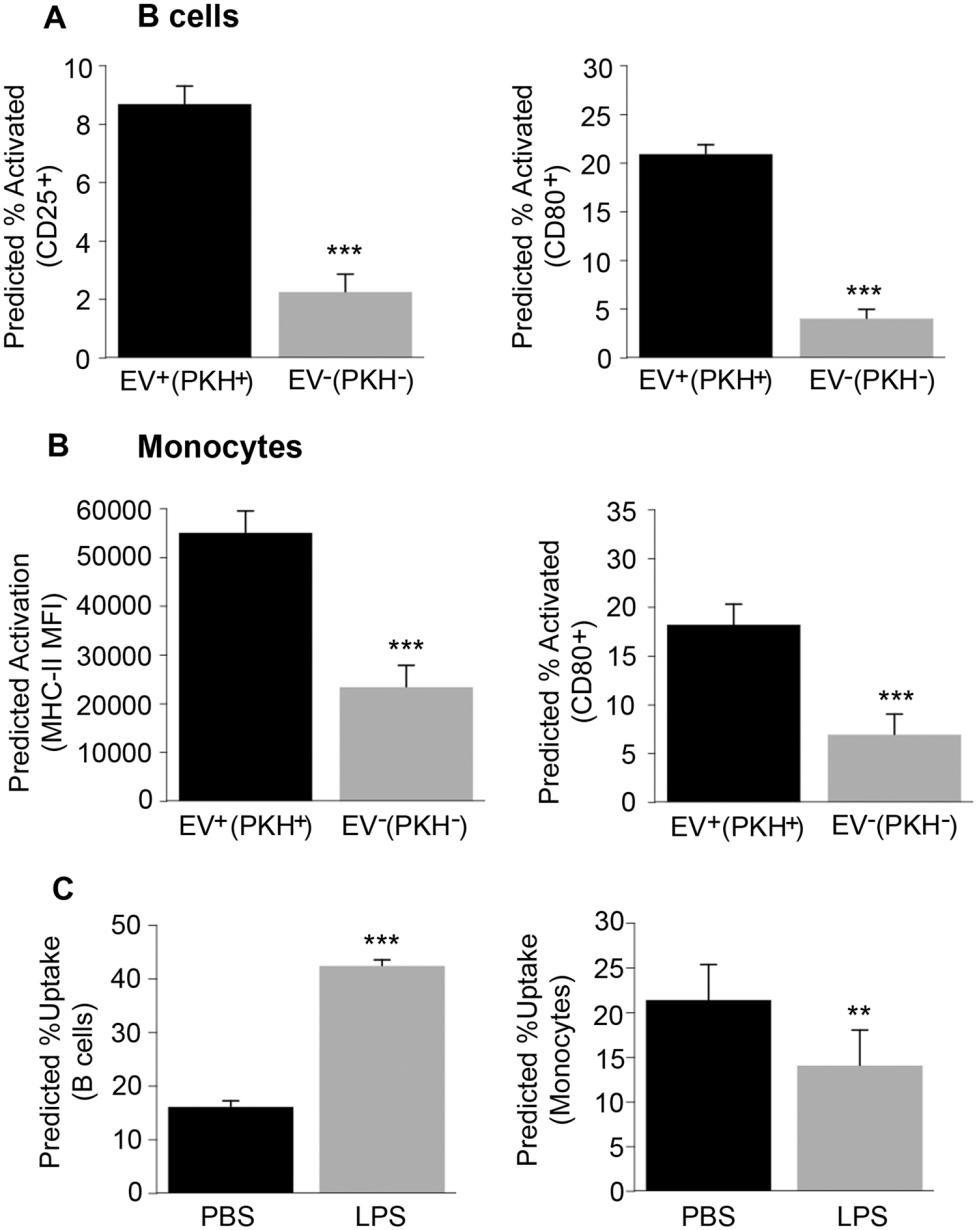
B cell and monocyte activation and EVs. (**A**) Plasma EVs (n = 54 EV donors) from young, middle-aged and older individuals were incubated with a different PBMC donor for 24 hrs and analyzed by FACS. (**B**) cells (CD19^+^) and monocytes (CD11b^+^CD14^+^CD15^−^) were stained with activation markers CD80, CD25 and MHC-II. B cell percentage that express CD25 or CD80 and were positive for EV uptake (CD19^+^PKH^+^CD25^+^ or CD19^+^PKH^+^CD80^+^) were compared to the B cell percentage that express CD25 or CD80 but were not positive for EV uptake (CD19^+^PKH^−^CD25^+^ or CD19^+^PKH^−^CD80^+^). (**B**) Monocytes that express MHC-II or CD80 and interacted with EVs (CD11b^+^PKH^+^MHC-II^+^ or CD11b^+^PKH^+^CD80^+^) was compared to the monocyte percentage that express MHC-II but did not interact with EVs (CD11b^+^PKH^−^MHC-II^+^ or CD11b^+^PKH^−^CD80^+^). (**C**) PBMCs from above were treated with LPS or PBS for 2 hrs then EVs (n = 23 a subset of EV donors from A-B) were added for 24 hrs followed by FACS. B cell percentage is shown for (CD19^+^PKH^+^) and monocyte percentage for (CD11b^+^PKH^+^). The histograms are the value from 54 EV samples for A-B and 23 EV samples for C predicted from linear mixed model regression accounting for matching + 95% confidence interval. ***P < 0.001 and **P < 0.01.

We further tested whether EVs activate monocytes/B cells or if activated monocytes/B cells preferentially internalize EVs. Interestingly, CD80, CD25 and MHC-II were increased on both B cells and monocytes that internalized EVs. (Fig. 4A,B and Fig. S5). Similar results were observed from a total of 5 PBMC donors (Fig. 4 and Fig. S5). These data suggest that EVs may activate both B cells and monocytes. To determine whether activated PBMCs internalize EVs more readily, PBMCs were treated with lipopolysaccharide (LPS). We found that LPS treatment increased B cell but not monocyte EV internalization (Fig. 4C). This data suggests that EVs may transmit inflammatory signals to immune cells, which may in turn continue the inflammatory cascade. On the other hand, activated immune cells (B cells) will also more readily uptake EVs.

### EV protein changes with age

**Figure 5:**
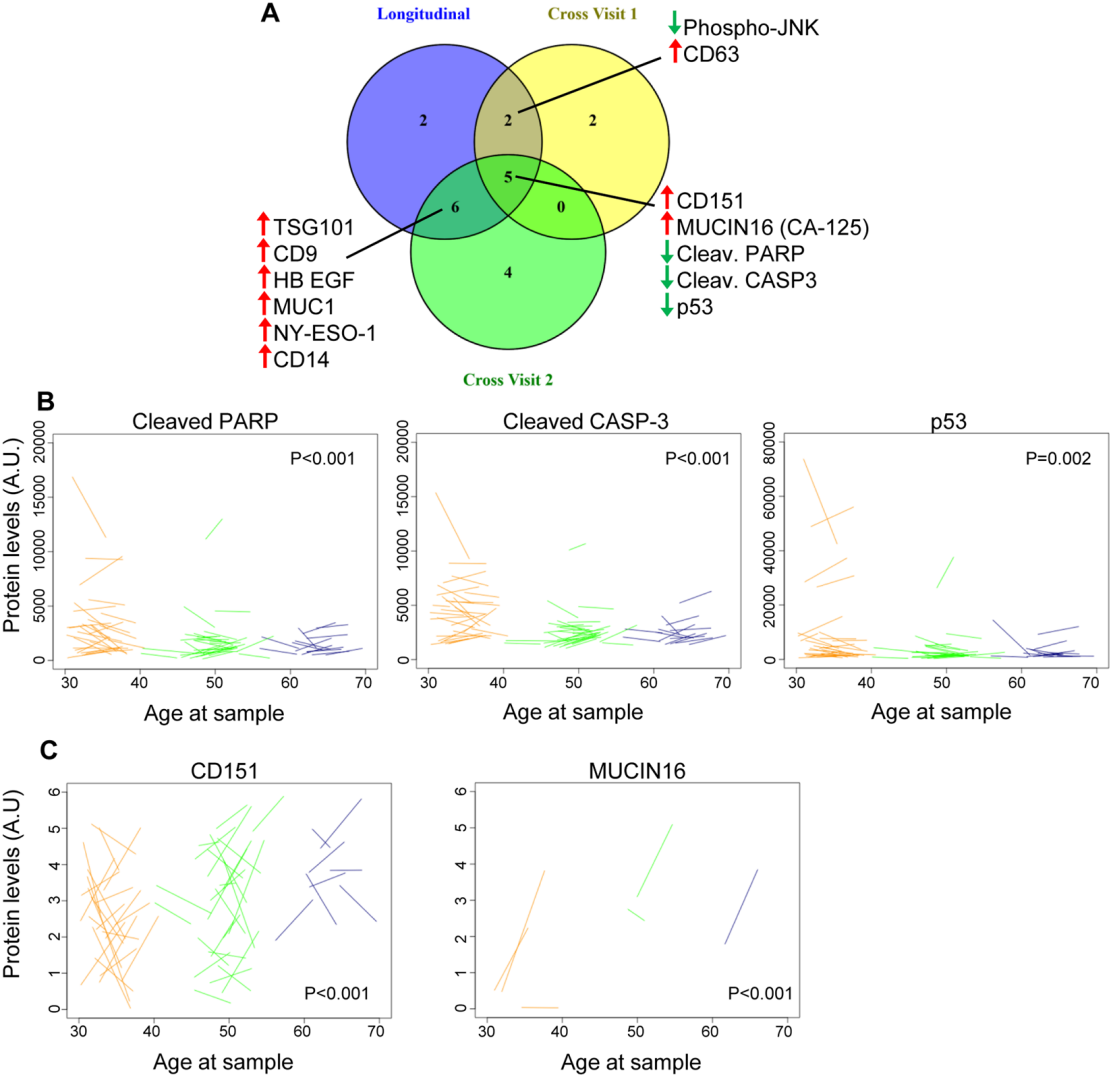
Significant changes in EV protein levels with age. (**A**) EV protein levels in 74 individuals at two different visits were quantified by EV Array or ELISA (see Methods for distinction). Linear mixed-effects models were used to determine whether EV protein levels changed significantly longitudinally (between visit 1 and visit 2) or in cross-sectional analysis of visit 1 or visit 2. Proteins that changed significantly in more than one analyses are listed and the direction of change is indicated by the up and down arrows. (**B**) Representative spaghetti plots for EV proteins that are significantly changed with age both in our longitudinal and cross-sectional analyses. The start of the line represents protein levels at time point 1 and the end of the line represents protein levels at time point 2. A.U. = arbitrary unit.

We hypothesized that vesicle proteins may account for the age-related differences in internalization and activation. We tested this idea using two different experimental approaches and measured protein levels from vesicles obtained from both visits. EV surface proteins were quantified using a recently developed assay called the EV Array^21^. Cytosolic proteins from lysed EVs were measured using ELISA assays. We found significant changes in protein levels in both our cross-sectional and longitudinal analyses. EV levels of several apoptosis markers decrease with age, including p53, cleaved PARP and cleaved Caspase-3 (Fig. 5). Two other proteins were significantly increased with age including CD151, a tetraspanin that enhances cellular processes involved in tumorigenesis and metastasis and MUCIN16 (also known as CA-125), a well-established circulating marker of ovarian cancer and possibly other types of cancers (Fig. 5). It should be noted that MUCIN16 was only detectable in 6 individuals at both visits and was detected in both males (n = 5) and one female (n = 1). There were additional individuals that had detectable EV MUCIN16 at visit 1 (n = 9) and visit 2 (n = 7) for our cross sectional analyses. Although MUCIN/CA-125 is predominantly expressed in female reproductive tissue and associated with reproductive cancers, it is also expressed in the lungs and gastrointestinal tract^22^, which may explain the presence of this protein in both males and females.

The most striking finding was the stability of each individual’s EV protein profile from visit 1 to visit 2. The levels of the majority of the proteins we examined were significantly correlated between visit 1 and visit 2 (Table 3). These data suggest that an individual’s EV protein profile is relatively consistent over the ~5 year time period assessed.

**Table 3:** Correlation of EV protein levels between visit 1 and visit 2.

Protein	r value	P value
AREG	0.73	<0.001
CD13	0.95	<0.001
CD14	0.76	<0.001
CD142	0.68	<0.001
CD146	0.55	0.001
CD151	0.57	<0.001
CD171	0.64	<0.001
CD206	0.61	<0.001
CD63	0.17	0.366
CD81	0.43	0.01
CD9	0.52	0.003
CEA	−0.35	0.055
Cleaved Caspase-3*	0.88	<0.001
Clusterin*	0.37	0.043
EGFR	0.59	<0.001
EGFRvIII	0.56	<0.001
EpCAM	0.68	<0.001
phospho-ERK1/2*	0.75	<0.001
Flotilin1	0.63	<0.001
HB EGF	0.69	<0.001
HER2	0.19	0.317
HER3	0.34	0.065
HER4	0.52	0.003
phospho-JNK*	0.8	<0.001
LC3	−0.11	0.558
MUC1	0.64	<0.001
Mucin16	0.62	<0.001
N-Cadherin	0.6	<0.001
NY-ESO-1	0.61	<0.001
Cleaved PARP*	0.89	<0.001
phospho-p38*	0.73	<0.001
p53	0.75	<0.001
p53*	0.9	<0.001
phospho-p53*	0.93	<0.001
phospho-p53/p53*	0.67	<0.001
PD-L1	0.51	0.004
PLAP	0.51	0.003
phospho-Tau181*	0.23	0.208
SFTPD	0.66	<0.001
SPA	0.61	<0.001
TAG72	0.8	<0.001
TNF RI	0.49	0.005
TNF RII	−0.06	0.764
TSG101	0.85	<0.001
Tspan8	0.53	0.002

EV proteins levels were analyzed by EV array or by ELISA (indicated by *). Protein levels between visit 1 and visit 2 were compared by Pearson correlation (r).

## Discussion

Although EVs were discovered over 5 decades ago^23^, only recently have data emerged that these vesicles play important roles in intercellular signaling and in regulating various physiological and pathological conditions^2^. EVs contain protein and RNA, and accumulating evidence indicates that EVs can provide molecular information on their cell of origin that can be utilized for diagnostic purposes termed a ‘liquid biopsy’^9^. Recently, numerous studies have investigated the potential of EVs to serve as biomarkers for various diseases including cancer, neurodegenerative, metabolic and immunologic disorders^2^. Such studies are designed to focus on comparisons between affected patients and age-matched controls. In contrast, the present study was designed to determine the impact of age on plasma EV phenotypes, and to elucidate potential influences of physiological and lifestyle factors on the characteristics of circulating EVs. Thus, we examined EVs in a cross-sectional and longitudinal manner among men and women who were either African American or white at two different study visits separated by ~5 years.

We found that the concentration of EVs in plasma decreased with age in both our cross-sectional analyses and in our longitudinal analysis. *In vitro* different cell types secrete different amounts of EVs and the levels are also dependent upon the cellular physiological conditions^24, 25^. For example, neuronal depolarization increases EV release^26^, and environmental stressors such as hypoxia increase EV release from breast cancer cells^27^. Higher concentration of EVs was also reported in the blood of cancer patients^18, 19^. We found that EV concentration was positively associated with BMI and smoking, which highlight the importance of follow-up studies focused on how lifestyle factors affect EV concentration. Recently, a potential cross-detection was observed between low-density lipoprotein (LDL) and EVs^28^. Although we cannot exclude that LDL may co-purify with EVs in our study, we did not find any significant relationships between EV concentration and LDL, HDL or cholesterol in our cohort using linear mixed model regression. Therefore, it is likely that the results we are observing our due to changes in EVs.

EV concentration reflects the balance between their secretion and internalization. EVs in the blood may interact with circulating cells and endothelial cells. Therefore, we chose to examine EV internalization by primary human PBMCs. To do this, we developed a FACS-based method that measured EV internalization and/or interaction with PBMCs. The internalization was dose- and time- dependent, which validates the assay sensitivity to detect differences in EV interactions with cells. We found that a high percentage of monocytes internalized EVs. B cells also internalized EVs, but T helper (CD4^+^) cells and effector T cells (CD8^+^) did not internalize detectable amounts of EVs. EV internalization significantly increased the proportion of B cells that express the general activation marker CD25 and CD80 and the proportion of monocytes expressing MHC-II and CD80. Therefore, interaction with EVs may influence monocyte antigen presentation, phagocytosis, T cell activation/suppressing ability, antimicrobial activity, or their role in tissue repair and angiogenesis, all of which change with aging^29^. On the other hand, EVs can also have an effect on B cell antibody production or modulate other B cell effector functions. While EVs isolated from the plasma of young and old individuals were internalized similarly with monocytes, EVs isolated from old individuals further enhanced expression of MHC-II. EV internalization by B cells was significantly enhanced with the age of the EV donor, but age of EVs did not influence B cell activation state. Interestingly, LPS-induced activation of B cells enhanced EV internalization. However, the same treatment did not increase monocyte EV internalization, which is consistent with other reports that LPS-treatment inhibits monocyte phagocytic activity^30^. Collectively, these results suggest that age can affect both EV internalization and activation of target cells, and that these differences are cell-type dependent. Moreover, at least part of the reduction in circulating EVs with age could be due to increased EV internalization by B cells. However, we cannot exclude that differences in EV secretion may also occur with age.

We examined EV surface protein levels using a newly developed method called the EV Array^21^, which measures the levels of 37 proteins on the EV surface. CD151 and MUCIN16 levels were increased with age in our cross-sectional analyses and in our longitudinal analysis, and CD9, CD14, MUC1 and NY-ESO1 were increased in the longitudinal and one of the cross-sectional analyses. In a recent study MUC1 levels were observed to decrease with age in plasma EVs in a small group of white males^31^. This difference further highlights the need for large clinical studies of EV alterations with age. CD9 and CD151 belong to the tetraspanin family, which regulate cell adhesion, motility, activation and proliferation^32^ and commonly used as exosome biomarkers. CD151 is highly expressed by platelets^33^, which may indicate that the different levels of CD151 with age may not be due to protein sorting into EVs but by changes in the composition of EVs that are released by different cell types. Levels of MUC1, MUCIN16, CD151 and NY-ESO1 are all reported to be higher in cancer cells^25, 34, 35^. These antigens have been reported to mediate immune evasion by cancer cells, and recently NY-ESO1 is an antigenic target for chimeric antigen receptor (CAR) T cells in a small clinical trial for multiple melanoma^36^. Furthermore, MUCIN16 is also referred to as CA-125 and is a well-established circulating marker of ovarian cancer and possibly other types of cancers. We found that PD-L1, a major target of anti-cancer immunotherapy^37^, was significantly increased in one of our cross-sectional analyses. Several other cancer-associated proteins, such as EGFRvIII, were present in EVs in our cohort. This may be surprising, but recently two other studies found that EGFRvIII was detectable in EVs from healthy individuals^31, 38^, which further indicates the need for further characterization of EV protein profiles under non-pathological conditions. It is also interesting to speculate that the increased expression of cancer antigens on the surface of EVs may be responsible for their elevated interaction with B cells.

In addition to measuring surface proteins by EV Array, we measured the levels of 9 cytosolic proteins by ELISA assays. We found that several markers of apoptosis including cleaved PARP1, cleaved Caspase-3 and p53 were reduced with age in both cross-sectional and longitudinal analyses. The levels of cleaved Caspase-3 were measurable, but relatively low, in EVs from most participants. The presence of these proteins in EVs may serve for signaling and/or may be a way for the EV-producing cells to expel the apoptosis promoting proteins^39^. It has been reported that EVs can mediate bystander effects of radiation treatment^40^, which is the transfer of the toxic effects of radiation from one cell to another. However, EVs were also reported to convey anti-apoptosis signals^41, 42^. It is therefore reasonable to speculate that the levels of these proteins in EVs may give information on the apoptotic status of their tissue of origin. This information may pass to circulating immune cells as part of their role in immune surveillance, and may be utilized as a biomarker for cells affected by disease.

Our cross-sectional and longitudinal mixed study design allows the ability to examine not only differences with age, but also identify changes with time. EV concentration for 39 out of 46 proteins showed a significant correlation between visits 1 and 2. This suggests that each individual has a relatively consistent level and type of EVs in their circulation. Such strong correlations between the two visits for both concentration and protein levels further validate the accuracy of our measurements. This is an important point, because very few studies have evaluated EVs isolated from samples taken from the same subjects after an extended time interval (~5 years in the present study). Our findings suggest that in cases when normalization to baseline is possible, such as when measuring response to treatment of a disease, EVs may provide non-invasive insight to evaluate disease progression. It may also prove to be valuable to generate an EV baseline as part of the clinical measurements associated with routine blood tests.

In addition to serving as biomarkers, EVs may have a role in systemic aging. Inflammation is a major hallmark of aging and EVs have a fundamental role in immune signaling^29, 43^. We found that several immune-related antigens like MUCIN16, MUC1, NY-ESO, CD14 and PDL increase with age and that EVs isolated from older individuals enhance monocyte activation. Loss of proteostasis is also an important hallmark of aging, and EVs have been suggested to participate in protein disposal. An example is the presence of many neurodegenerative disease-related misfolded proteins in EVs^44^. Therefore, the reduction in EV concentration may be a consequence of an impaired clearance mechanism that together with cell autonomous proteostasis malfunction promotes the accumulation of pathogenic proteins. The reduction in EV concentration can also be a consequence of aging-related phenotypic changes like cellular senescence^45^ or part of the altered intercellular signaling; both are important hallmarks of aging^29^. EVs may be part of the aging mechanism and may change as a consequence of aging-related mechanisms and thus serve as biological aging indicators. While the current study shows that EVs change with age, further research is required to clarify their role in aging.

In conclusion, our results clearly show that EV concentration and protein composition change with age. Furthermore, our data indicate that individuals may have a specific EV concentration and protein profile. These data provide important insight into the characterization of EVs in non-pathological conditions that may enhance the utility of EVs to serve as biomarkers for diagnosis, prognosis and pharmacological responses to therapeutic intervention.

## Materials and Methods

### Cohort Design and Plasma Isolation

Participants for this study were selected from the Healthy Aging in Neighborhoods of Diversity across the Life Span (HANDLS) study of the National Institute on Aging Intramural Research Program (NIA IRP) of the National Institutes of Health (NIH). HANDLS is a longitudinal, epidemiologic study designed to examine the effect of socioeconomic status, race, and gender on aging and health to enhance our understanding of health disparities. We chose a total of 74 participants from the following groups: young (30–40), middle aged (45–55) and old (55–65). Each group was race and sex matched. The cohort included both Whites and African Americans. Body mass index (BMI = weight [kg]/height [m^2^]) was calculated from measured height and weight. Smoking use was defined as current or non-user. Data was collected during a structured medical history interview and a physical examination. More details on the demographics and clinical characteristics of the cohort can be found in Table 1. For each participant, we chose 2 samples that were obtained ~5 years apart (mean = 4.60 ± 1.04 years). Participants diagnosed with cancer, Alzheimer’s disease, HIV or Hepatitis B/C were excluded from the cohort. All participants provided written informed consent and the study has been approved by the Institutional Review Board (IRB) of the National Institute of Environmental Health Sciences (NIEHS), NIH. All experiments were performed in accordance with relevant guidelines and regulations.

### Plasma EV Isolation

A fasting blood sample was collected between 9:30–11:00 a.m. using a 20-gauge butterfly from each participant into EDTA blood collection tubes. EDTA tubes were centrifuged at 600 × *g* for 15 min with the brake on, then the buffy coat was removed. These steps were repeated a total of two times. Samples are visually inspected for hemolysis. Plasma samples were immediately aliquoted and stored at −80 °C. Extracellular vesicles (EVs) were isolated from 0.45 mL of plasma, thawed on ice, using ExoQuick™ Exosome Precipitation Solution (System Bioscience Inc.) with some modifications from the manufacturer’s protocol^8^. Plasma was treated with 0.15 mL Thromboplastin D (Cat#:100354; Fisher Scientific, Inc.), incubated at room temperature for one hr and then 0.35 mL of Dulbecco’s phosphate buffered saline (DPBS) was added. Fibrin proteins were separated by centrifugation at 3000 × *g* for 20 min at 4 °C. The supernatants were collected and mixed with ExoQuick™, incubated for one hour at 4 °C and then centrifuged at 1500 × *g* at 4 °C for 20 min. The supernatant was removed and saved for analysis as the EV depleted plasma fraction. The EV pellet was centrifuged again at 1500 × *g* at 4 °C for 10 minutes to remove all liquid. The pellet was resuspended in 0.5 mL of nanopure water^46^. Protease and phosphatase inhibitor cocktails were present throughout the protocol (Roche Applied Sciences). An aliquot of the EV fraction was then diluted in DPBS at a 1:300 dilution for enumeration and the remaining was stored at −80 °C until further analysis.

For the ultracentrifugation method, plasma samples (200 μl) were diluted in sterile PBS up to 4.5 ml in a Beckman ultracentrifugation tube and then centrifuged at 120,000 × *g* for 2 hours (SW28 rotor K = 246). The supernatant was carefully removed, sterile PBS was added and the centrifugation was repeated. After the second centrifugation, the supernatant was carefully removed and the pellet was suspended in 100 μl of PBS by repeated pipetting. The sample was diluted 1:100 and stored in −80 until quantification by NTA analysis.

For the qEVmethod, the qEV size exclusion columns were from iZON Science and the EVs were isolated according to the manufacturer’s procedure.

### Immunoblotting

EVs from plasma were thawed and lysed by adding 3:1 volume of M-PER™ Reagent (Thermo Scientific), supplemented with protease and phosphatase inhibitors (Roche). Lysates were vortexed for 30 secs. 5 µg of the samples, U87 glioblastoma cell line lysate sample, and two ExoQuick™ EV-depleted supernatant samples were analyzed by SDS-PAGE followed by immunblotting with antibodies against EV protein markers: CD9 (clone H-110, Santa Cruz Biotechnologies Inc.), Flotillin 1 (clone EPR6041, Abcam, UK) and TSG101 (clone EPR7131(B), Abcam, UK).

### Electron Microscopy

EVs were allowed to adsorb to freshly ionized 300 mesh formvar/carbon coated grids then washed briefly through 5–7 puddles of ddH_2_O and subsequently negatively stained in 2% aqueous uranyl acetate. Images were taken with transmission electron microscopy (FEI Tecnai G2 Spirit) with TWIN Lens operating at 100 kV and using an Olympus Soft Imaging System Megaview III digital CCD.

### Nanoparticle Tracking Analysis

EV size and concentration were determined by nanoparticle-tracking analysis using a NanoSight NS500 (Malvern Instruments Ltd.) as instructed by the manufacturer’s protocol. For each sample, five 20 second videos were recorded at Camera Level = 11. Analysis was performed at Detection Limit = 3. All samples were analyzed at the same settings to enhance concentration measurement accuracy^47^. EV isolation and size and concentration analyses were performed blind and samples from visit 1 and visit 2 were processed at the same time. The plasma EV concentration was calculated using the following equation:

$$\begin{array}{c}PlasmaEVConcentation=\frac{NTAoutput(Particle/mL)\ast DilutionFactor\ast EVSampleVolume}{StartingPlasmaVolume}\end{array}$$

### PBMC EV Internalization

Human peripheral blood was collected by the Health Apheresis Unit and the Clinical Core Laboratory, the National Institute on Aging, under Human Subject and Tissue Procurement Protocols. All participants provided written consent and the protocols have been approved by the IRB of the NIEHS, NIH. Peripheral blood mononuclear cells were isolated using Ficoll-Paque (GE Healthcare) density gradient separation according to the manufacturer’s instruction. PBMCs from five donors ranging in age from 23–59 (mean = 42.4 + 13.78 yrs) were used for experiments in Fig. 2. Three were male and 2 were female, 2 were smokers and none had allergies. PBMCs from four donors were used for Fig. 3 and Fig. S3–S5, two young (34, 26 yrs) and two old (82, 84 yrs). For Fig. 4, PBMCs from a 45 yr old female were used.

Plasma EVs were labeled with 0.1 μM (f.c.) PKH26 (Cat#1077 Sigma-Aldrich, St. Louis, MO) for 10 min. The sample was then centrifuged through an Exosome Spin Column MW (Life Technologies-Invitrogen) to remove unincorporated dye according to the manufacturer’s instructions. The samples were then enumerated using the NanoSight NS500 as described above. PKH26 labeled EVs were then added to PBMCs in the doses between 2.00^E+08^–5.75^E+09^ particles. The PBMCs and EVs were then incubated for 1–24 hours in RPMI complete media (RPMI, 10% FBS, 1% Penicillin/Streptomycin/Glutamine, 0.1% Sodium Pyruvate, 0.1% HEPES, 1% MEM Non-essential amino acids solution, 0.1% 2-Mercaptoethanol; all from Invitrogen) before FACS. PBMCs were also incubated with Latrunculin A (1 ng/mL), an endocytosis inhibitor, for 30 minutes before the PBMCs received a dose of PKH labeled EVs. PBMCs were treated with LPS (500 ng/ml; from Sigma-Aldrich) for 2 hrs and then incubated with EVs for 24 hrs. All experiments were performed with approximately 200,000 PBMCs.

### FACS analysis

After incubation with EVs, the PBMCs were washed three times in FACS buffer (0.5% BSA in PBS) and then stained with fluorescent labeled monoclonal antibodies from Biolegend: APC anti-human CD19 (clone HIB19), FITC anti-mouse/human CD11b (clone M1/70), FITC anti-human CD14 (clone M5E2), PerCP/Cy5.5 anti-human CD25 Antibody (clone BC96), PE/Cy7 anti-human HLA-DR Antibody (clone L243), and Pacific Blue™ anti-human CD3 (clone HIT3a) or PerCP/Cy5.5 anti-CD4 (clone OKT4), and PE/cy7 anti-human CD8 (clone RPA-T8), according to manufacturer’s recommendations. In Fig. 4, we used additional antibodies including BV422 anti-human CD80 (clone 2D10), Alexa Fluor 700 CD15 (cloneW6D3), FITC anti-human CD14 (M5E2), and AmCyan CD11b (clone M1/70). After antibody incubation, cells were washed two times in FACS buffer. Flow cytometry data was collected on a FACS Canto II (BD, Franklin Lakes, NJ). Dead cells were excluded from analysis using forward and sideward scattering gating. Live cells were gated based on cell surface markers CD11b, CD14, CD19, CD4 and CD8 or CD3 to identify monocytes, B cells, and T cells, respectively. For Fig. 4, percent monocytes are shown as CD14^+^CD15^−^ cells gated on CD11b^+^ population. The cell populations were then also gated for PKH to identify cells that internalized the labeled EVs. The flow cytometry data was analyzed with FlowJo software (Tree Star, Inc.).

### ELISA

Plasma EVs from above were lysed using M-PER™ Reagent (Thermo Scientific), supplemented with protease and phosphatase inhibitors (Roche). Equal volume of EV lysates were used to quantitatively measure proteins in the following Meso Scale kits (Meso Scale Diagnostics): total p53, phospho-p53 (Ser15), PARP (cleaved), and Caspase-3 (cleaved) by the Apoptosis Whole Cell Lysate Kit (Cat#K15102D-1); phospho-JNK (Thr183/Tyr185), phospho-p38 (Thr180/Tyr182), and phospho-ERK1/2 (Thr/Tyr: 202/204; 185/187) by the Apoptosis Whole Cell Lysate Kit (Cat# K15101D-1) and a custom made kit for phospho-Tau181 kit. Meso Scale kits were analyzed on a Meso Quickplex SQ 120. LC3 was measured using an ELISA kit (Cat# MB-S2602917 MyBioSource, Inc.). Clusterin was quantified by the Clusterin Competitive ELISA Kit (Cat# AG-45A-0013YEK-KI01 Adipogen Corporation).

### EV Array

EV surface protein markers were quantified by Extracellular Vesicle Array analysis^21^. To capture EVs from plasma, a microarray print with spots of 37 different antibodies were used to capture EVs, then detected using a cocktail of biotinylated antibodies against the tetraspanins (CD9, CD63 and CD81) followed by detection with fluorescent-streptavidin conjugated antibodies^21, 38^. Plasma (10 µl) from 74 individuals at the two different time points was used for analysis (see Table 1 for demographics). The EV array included 37 proteins associated with EVs, cancer, neuronal, endothelial or/and immune protein markers. All proteins are listed in Table 3 except for c-Met, which was excluded since it was only detectable in 7 participants. Zero values were excluded from our analysis.

### Statistics

Statistical analyses were performed using R software version 3.3.0. Differences among demographic variables were analyzed using Pearson’s chi-square tests for categorical variables and one-way ANOVA for continuous variables. EV concentration, internalization and protein models, either cross-sectional or longitudinal, were analyzed using linear mixed model regression accounting for the matched design by race and sex. Significance of fixed factors was determined by log likelihood tests. Correlations between visit 1 and 2 for EV concentration and protein levels were assessed by Pearson correlation coefficients (r) with degrees of freedom accounting for the matching by race and sex. Internalization values for B cells, T cells and monocytes were strongly positively skewed and thus were log transformed for linear mixed model analysis accounting for matching by race and sex.

## Electronic supplementary material

Supplementary Information

## References

[1] Colombo M, Raposo G, Thery C (2014). Biogenesis, secretion, and intercellular interactions of exosomes and other extracellular vesicles. Annu Rev Cell Dev Biol.

[2] Yanez-Mo M (2015). Biological properties of extracellular vesicles and their physiological functions. J Extracell Vesicles.

[3] Lotvall J (2014). Minimal experimental requirements for definition of extracellular vesicles and their functions: a position statement from the International Society for Extracellular Vesicles. J Extracell Vesicles.

[4] Kanada M, Bachmann MH, Contag CH (2016). Signaling by Extracellular Vesicles Advances Cancer Hallmarks. Trends in Cancer.

[5] Smith, J. A. *et al*. Extracellular vesicles and their synthetic analogues in aging and age-associated brain diseases. *Biogerontology*, 1–39, 10.1007/s10522-014-9510-7 (2014). 10.1007/s10522-014-9510-7 PMC4578234 24973266

[6] Arraud N (2014). Extracellular vesicles from blood plasma: determination of their morphology, size, phenotype and concentration. J Thromb Haemost.

[7] Nielsen MH, Beck-Nielsen H, Andersen MN, Handberg A (2014). A flow cytometric method for characterization of circulating cell-derived microparticles in plasma. J Extracell Vesicles.

[8] Fiandaca MS (2015). Identification of preclinical Alzheimer’s disease by a profile of pathogenic proteins in neurally derived blood exosomes: A case-control study. Alzheimers Dement.

[9] Santiago-Dieppa DR (2014). Extracellular vesicles as a platform for ‘liquid biopsy’ in glioblastoma patients. Expert Rev Mol Diagn.

[10] Turpin D (2016). Role of extracellular vesicles in autoimmune diseases. Autoimmun Rev.

[11] Al-Nedawi K (2008). Intercellular transfer of the oncogenic receptor EGFRvIII by microvesicles derived from tumour cells. Nature cell biology.

[12] Verma M, Lam TK, Hebert E, Divi RL (2015). Extracellular vesicles: potential applications in cancer diagnosis, prognosis, and epidemiology. BMC Clin Pathol.

[13] Hunter MP (2008). Detection of microRNA Expression in Human Peripheral Blood Microvesicles. PloS one.

[14] Andreu, Z. *et al*. Comparative analysis of EV isolation procedures for miRNAs detection in serum samples. 2016 (2016). 10.3402/jev.v5.31655 PMC4916259 27330048

[15] Kordelas L (2014). MSC-derived exosomes: a novel tool to treat therapy-refractory graft-versus-host disease. Leukemia.

[16] Helwa I (2017). A Comparative Study of Serum Exosome Isolation Using Differential Ultracentrifugation and Three Commercial Reagents. PloS one.

[17] Dragovic RA (2011). Sizing and phenotyping of cellular vesicles using Nanoparticle Tracking Analysis. Nanomedicine: Nanotechnology, Biology and Medicine.

[18] Gercel-Taylor C, Atay S, Tullis RH, Kesimer M, Taylor DD (2012). Nanoparticle analysis of circulating cell-derived vesicles in ovarian cancer patients. Anal Biochem.

[19] Rodriguez M (2014). Different exosome cargo from plasma/bronchoalveolar lavage in non-small-cell lung cancer. Genes Chromosomes Cancer.

[20] Thery C, Ostrowski M, Segura E (2009). Membrane vesicles as conveyors of immune responses. Nat Rev Immunol.

[21] Jorgensen M (2013). Extracellular Vesicle (EV) Array: microarray capturing of exosomes and other extracellular vesicles for multiplexed phenotyping. J Extracell Vesicles.

[22] Uhlén M (2015). Tissue-based map of the human proteome. Science.

[23] Wolf P (1967). The nature and significance of platelet products in human plasma. Br J Haematol.

[24] Sokolova V (2011). Characterisation of exosomes derived from human cells by nanoparticle tracking analysis and scanning electron microscopy. Colloids Surf B Biointerfaces.

[25] Soo CY (2012). Nanoparticle tracking analysis monitors microvesicle and exosome secretion from immune cells. Immunology.

[26] Lachenal G (2011). Release of exosomes from differentiated neurons and its regulation by synaptic glutamatergic activity. Molecular and cellular neurosciences.

[27] King HW, Michael MZ, Gleadle JM (2012). Hypoxic enhancement of exosome release by breast cancer cells. BMC Cancer.

[28] Sódar BW (2016). Low-density lipoprotein mimics blood plasma-derived exosomes and microvesicles during isolation and detection. Scientific reports.

[29] Lopez-Otin C, Blasco MA, Partridge L, Serrano M, Kroemer G (2013). The hallmarks of aging. Cell.

[30] Feng X (2011). Lipopolysaccharide inhibits macrophage phagocytosis of apoptotic neutrophils by regulating the production of tumour necrosis factor α and growth arrest-specific gene 6. Immunology.

[31] Bæk R, Varming K, Jørgensen MM (2016). Does smoking, age or gender affect the protein phenotype of extracellular vesicles in plasma?. Transfusion and Apheresis Science.

[32] Bari R (2011). Tetraspanins regulate the protrusive activities of cell membrane. Biochem Biophys Res Commun.

[33] Orlowski E (2009). A platelet tetraspanin superfamily member, CD151, is required for regulation of thrombus growth and stability *in vivo*. J Thromb Haemost.

[34] Chauhan SC (2006). Aberrant expression of MUC4 in ovarian carcinoma: diagnostic significance alone and in combination with MUC1 and MUC16 (CA125). Mod Pathol.

[35] Kumari S, Devi Gt, Badana A, Dasari VR, Malla RR (2015). CD151-A Striking Marker for Cancer Therapy. Biomark Cancer.

[36] Rapoport AP (2015). NY-ESO-1-specific TCR-engineered T cells mediate sustained antigen-specific antitumor effects in myeloma. Nature medicine.

[37] Powles T (2014). MPDL3280A (anti-PD-L1) treatment leads to clinical activity in metastatic bladder cancer. Nature.

[38] Jakobsen KR (2015). Exosomal proteins as potential diagnostic markers in advanced non-small cell lung carcinoma. J Extracell Vesicles.

[39] Eitan E, Suire C, Zhang S, Mattson MP (2016). Impact of Lysosome Status on Extracellular Vesicle Content and Release. Ageing Research Reviews.

[40] Jella KK (2014). Exosomes are involved in mediating radiation induced bystander signaling in human keratinocyte cells. Radiat Res.

[41] Rani S, Ryan AE, Griffin MD, Ritter T (2015). Mesenchymal Stem Cell-derived Extracellular Vesicles: Toward Cell-free Therapeutic Applications. Mol Ther.

[42] Yang L, Wu XH, Wang D, Luo CL, Chen LX (2013). Bladder cancer cell-derived exosomes inhibit tumor cell apoptosis and induce cell proliferation *in vitro*. Mol Med Rep.

[43] Greening DW, Gopal SK, Xu R, Simpson RJ, Chen W (2015). Exosomes and their roles in immune regulation and cancer. Semin Cell Dev Biol.

[44] Janas AM, Sapon K, Janas T, Stowell MH, Janas T (2016). Exosomes and other extracellular vesicles in neural cells and neurodegenerative diseases. Biochimica et biophysica acta.

[45] Lehmann BD (2008). Senescence-associated exosome release from human prostate cancer cells. Cancer Res.

[46] Kapogiannis D (2015). Dysfunctionally phosphorylated type 1 insulin receptor substrate in neural-derived blood exosomes of preclinical Alzheimer’s disease. FASEB J.

[47] Gardiner C, Ferreira YJ, Dragovic RA, Redman CW, Sargent IL (2013). Extracellular vesicle sizing and enumeration by nanoparticle tracking analysis. J Extracell Vesicles.

