# Supplementary material for: Age-Related Changes in Plasma Extracellular Vesicle Characteristics and Internalization by Leukocytes
Source: Sci Rep. 2017 May 2;7:1342. doi: 10.1038/s41598-017-01386-z (PMC5430958; doi:10.1038/s41598-017-01386-z)
Supplement: Supplementary file 1 — Supplementary Information [file 41598_2017_1386_MOESM1_ESM.pdf]

<u>Supplementary Information: Figures S1-S5</u>

Age-Related Changes in Plasma Extracellular Vesicle Characteristics and Internalization by Leukocytes

Erez Eitan, Jamal Green, Monica Bodogai, Nicolle A. Mode, Rikke Bæk, Malene M. Jørgensen, David W. Freeman, Kenneth W. Witwer, Alan B. Zonderman, Arya Biragyn, Mark P. Mattson, Nicole Noren Hooten and Michele K. Evans

Figure S1

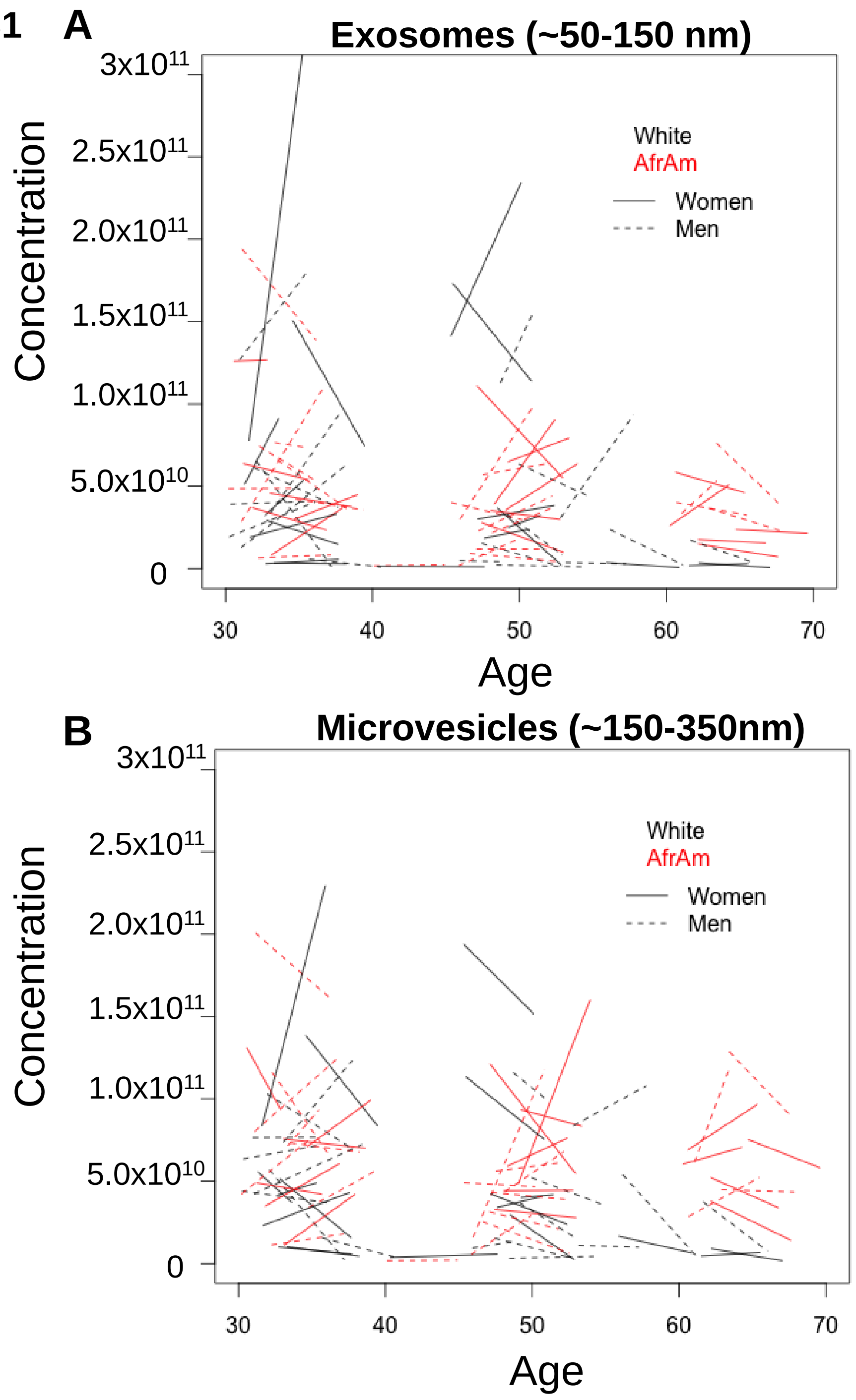

Figure S1. Changes in EV size over time. EV sizes from 74 individuals at two different time points were quantified using NTA. EVs were divided into categories based on size as either (A) exosomes (~50-150nm) or (B) microvesicles (~150-300 nm). Lines represent values for women (solid) and men (dashed) as well as African American (red lines) and White (black lines) participants. The start of the line is the value at Visit 1 and the end of the line is the value at Visit 2. The length of the line represents the amount of time between visits and the slope of the line represents how much the concentration changed over the time period. No significant changes in EV size were observed over time.

Figure S2

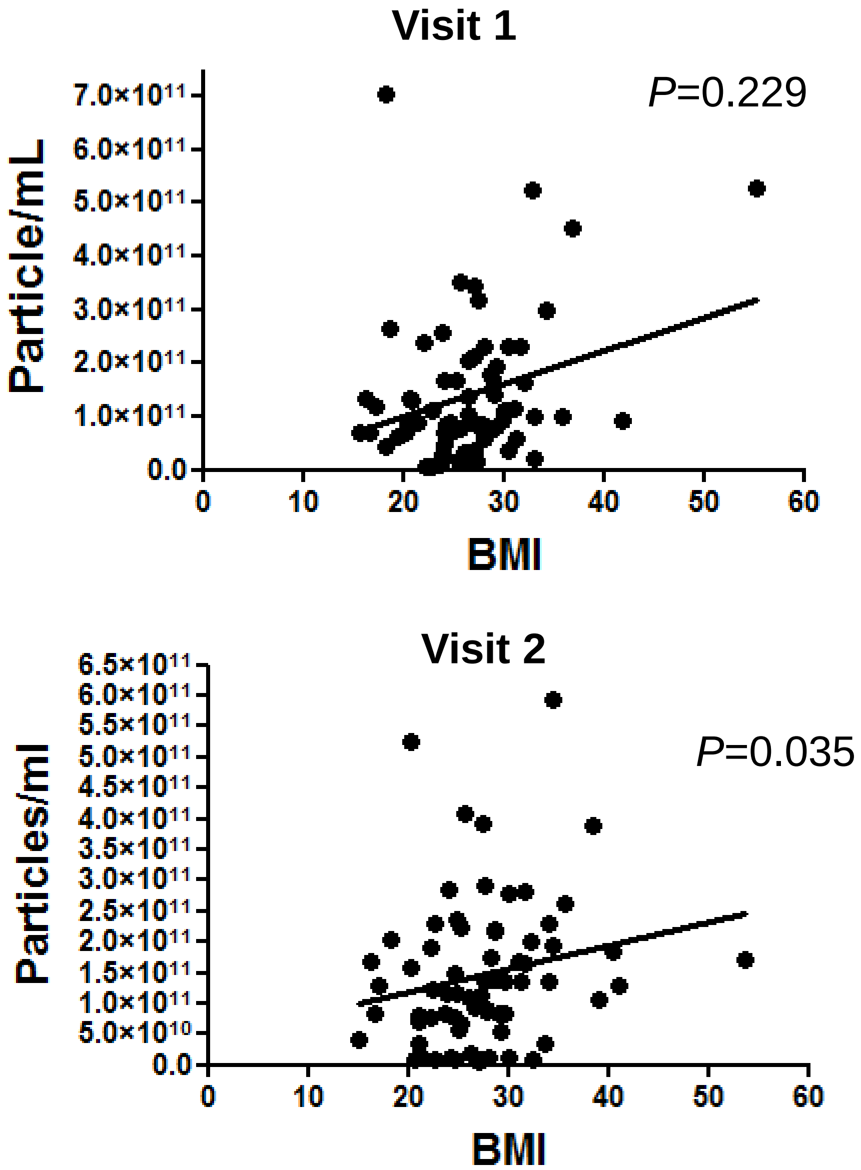

Figure S2. Association of BMI with EV concentration. (A) EV concentration (particles/ml) from young, middle-aged and older individuals (n=70) from visit 1 and visit 2 were quantified by NTA. Particle concentration was plotted against BMI. *P* values were determined by linear mixed-model regression.

Figure S3

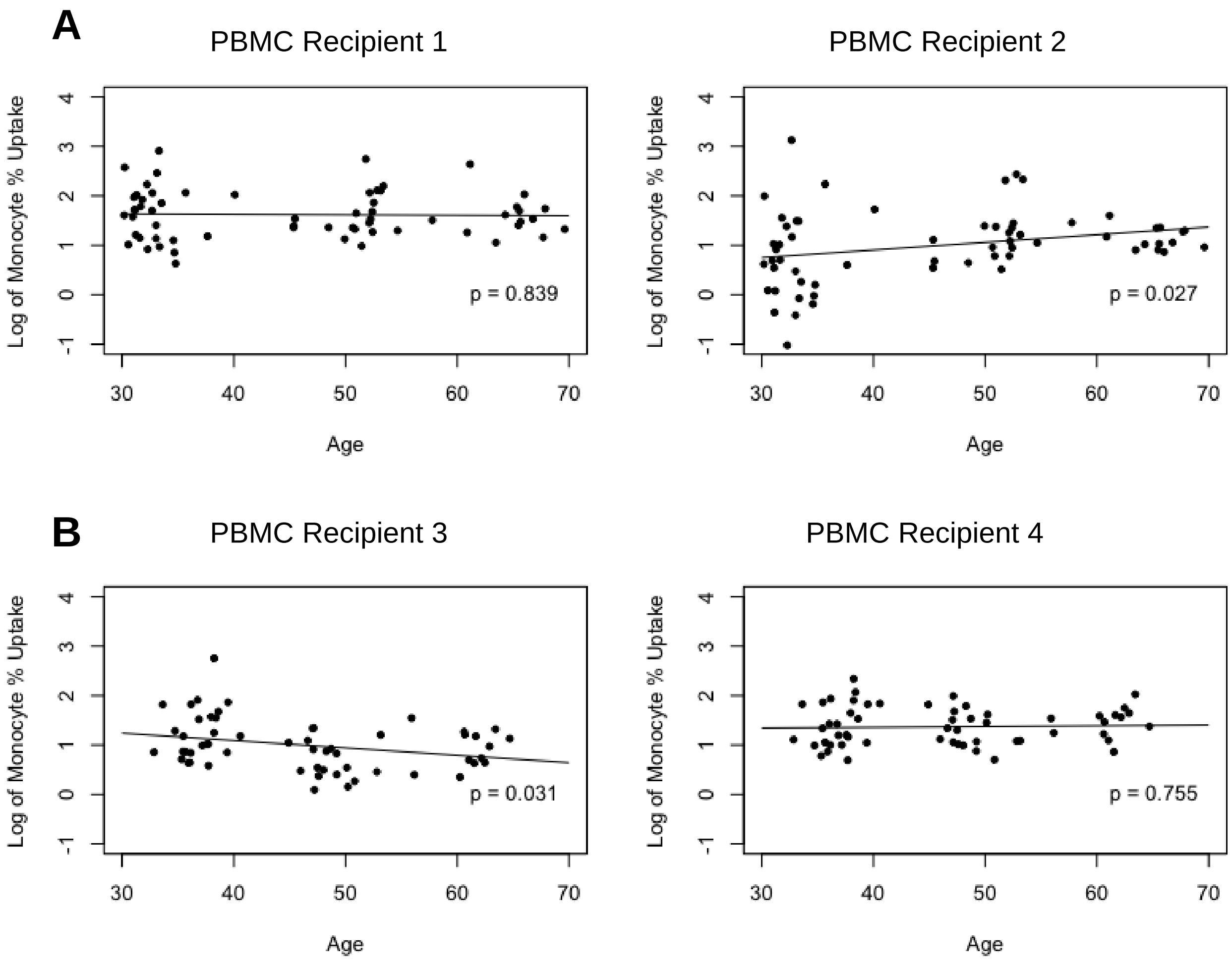

Figure S3. Monocyte internalization of EVs from young, middle-aged and older individuals. (A) EV plasma samples (n=62; 2.00^{E+08} particles) from young, middle-aged and older individuals were incubated with 1 old (1) and 1 young (2) PBMC donor. (B) In a separate experiment, EV plasma samples (n=57) from young, middle-aged and older individuals were incubated with a different old (3) and young (4) PBMC donor. In both experiments, EVs were incubated with PBMCs for 24 hours and then analyzed by FACS. Linear mixed model regression was used to analyze the relationship of EV donor age and monocyte internalization accounting for both sex and race matching. The points are the actual data (log transformed) and the line is from the model accounting for matching.

Figure S4

A B cells

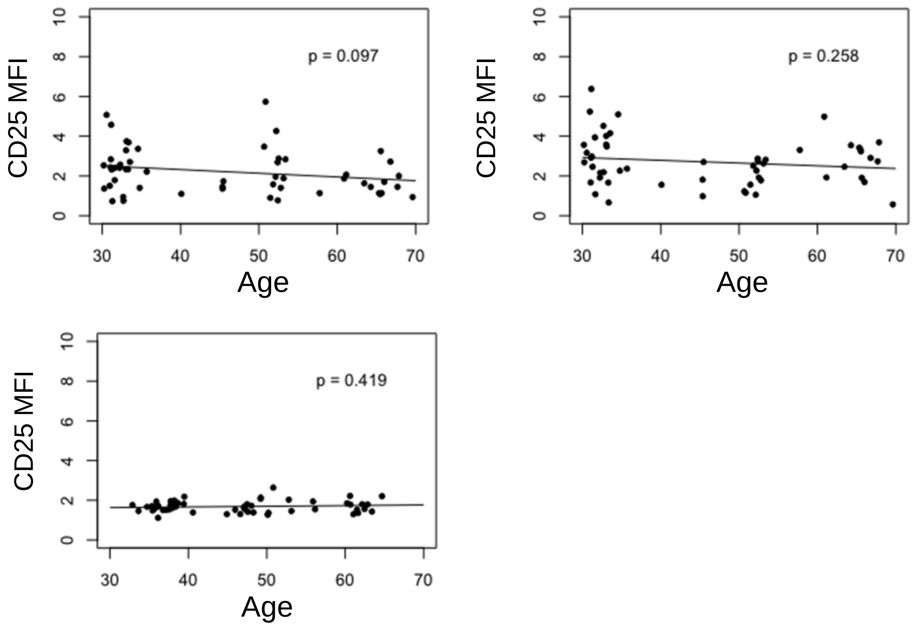

B Monocytes

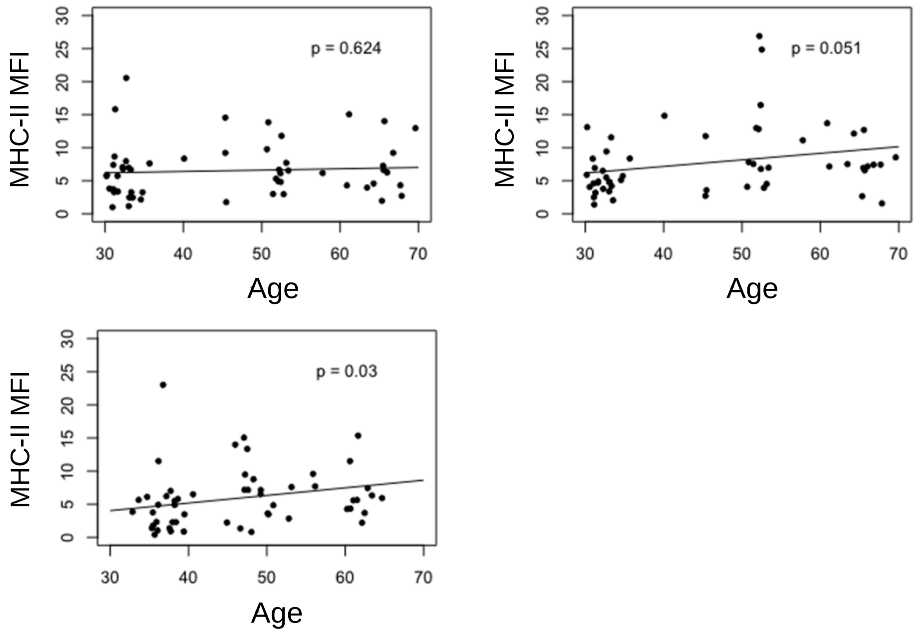

Figure S4. Effects of EV internalization on B cell and monocyte activation markers with age. Plasma EVs were incubated with PBMCs and stained for B cell and monocyte activation markers CD25 and MHC-II, respectively and for EV internalization (PKH^+). (A) The level of CD25 expression in B cells that interacted with EVs ($CD19^+PKH^+CD25^+$) were plotted against the age of the EV donor. (B) The level of MHC-II expression in monocytes that interacted with EVs ($CD11^+PKH^+MHC\text{-}II^+$) were plotted against the age of the EV donor. The scatter plots are 62 EV donors for PBMC recipient 1 and 2 and 57 EV donors for PBMC recipient 4. Linear mixed model regression was used to analyze the relationships accounting for both sex and race matching and *P* values are indicated. MFI, mean fluorescence intensity.

Figure S5

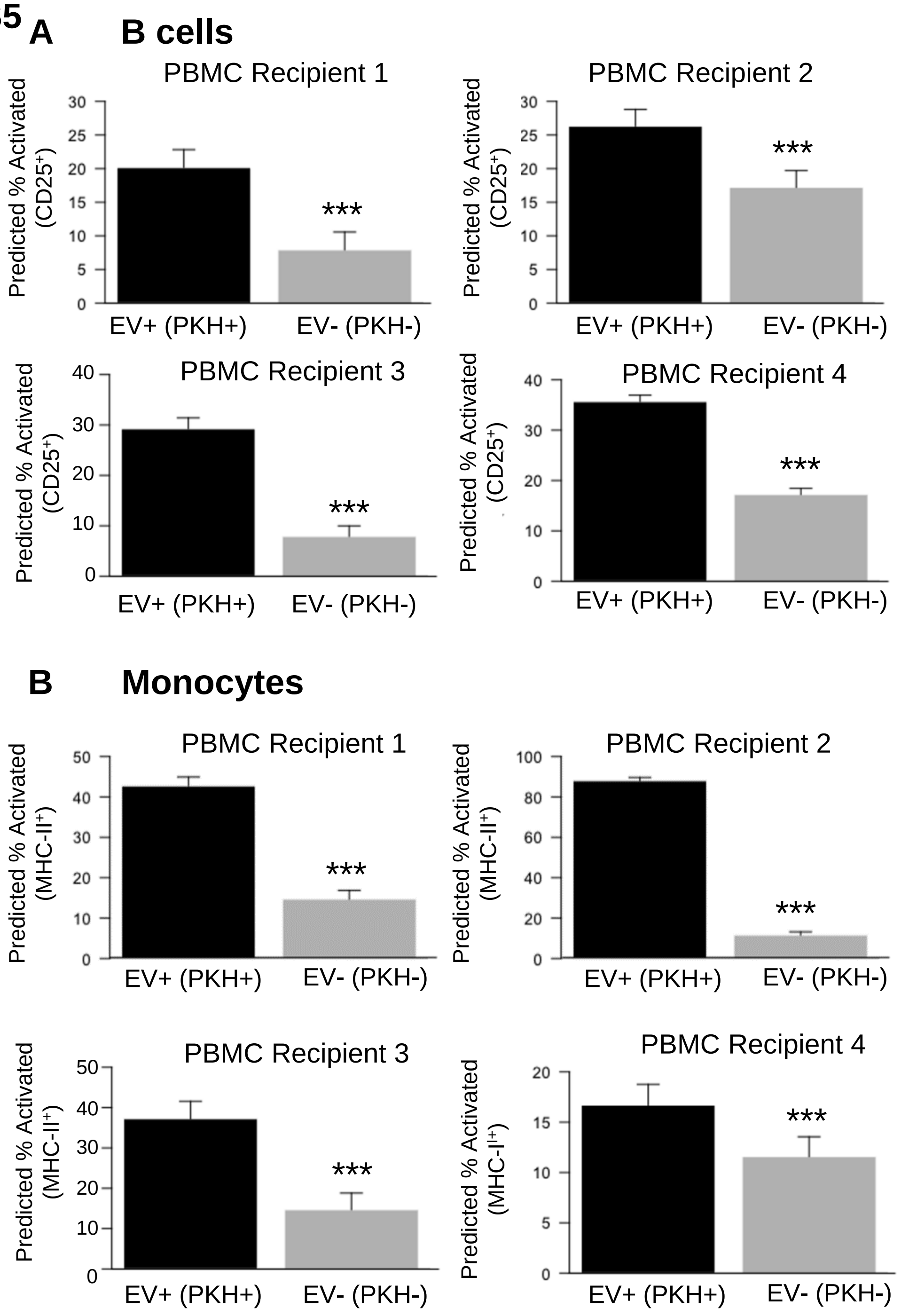

Figure S5. EV internalization increases the number of activated B cells and monocytes. Plasma EVs were incubated with PBMCs and stained for B cell and monocyte activation markers CD25 and MHC-II, respectively and for EV internalization (PKH^+). (A) The percentage of B cells that express the activation marker CD25 and were positive for uptake of EVs ($CD19^+PKH^+CD25^+$) was determined and compared to the percentage of B cells that express CD25 but were not positive for EV uptake ($CD19^+PKH^-CD25^+$). (B) The percentage of monocytes that express MHC-II and interacted with EVs ($CD11^+PKH^+MHC\text{-}II^+$) was determined and compared to the percentage of monocytes that express MHC-II but did not interact with EVs ($CD11^+PKH^-MHC\text{-}II^+$). The histograms are the value from 62 EV samples for PBMC recipient 1 and 2 and 57 EV samples for PBMC recipient 3 and 4 predicted from linear mixed model regression + 95% confidence interval. Linear mixed model regression was used to analyze the relationships accounting for both sex and race matching and ****P*<0.001.
